# Respiratory syncytial virus in pediatric patients admitted to a tertiary center in Amman: clinical characteristics, and age-related patterns

**DOI:** 10.1186/s12887-024-04799-8

**Published:** 2024-05-15

**Authors:** Montaha Al-Iede, Abdullah Alhouri, Khaled Marwa, Roaa Alnajjar, Mohammad Abuzenah, Bilala Abu-Hussein, Shereen M. Aleidi, Enas AL-Zayadneh, Amirah Daher, Basim Alqutawneh, Lena Sarhan

**Affiliations:** 1 Division of Pediatric Pulmonology and Sleep Medicine, Department of Pediatrics, Jordan University Hospital, Amman, Jordan; 2 The School of Medicine, The University of Jordan, Queen Rania Street, Amman, 11942 Jordan; 3 Division of Respiratory Medicine, Department of Medicine, Nevill Hall Hospital, Aneurin Bevan University Health Board, Wales, UK; 4 Division of Stroke, Department of Medicine, University Hospital Southampton, Southampton, UK; 5 Faculty of Pharmaceutical Sciences, The University of Jordan , Amman, Jordan; 6 Neurosurgery Department, Sheffield Teaching Hospital, NHS Foundation Trust, Sheffield, England; 7 Department of General Surgery, North Cumbria Integrated Care, Carlisle, UK; 8 Department of Biopharmaceutical and Clinical Pharmacy, School of Pharmacy, The University of Jordan, Amman, Jordan; 9 Division of Intensive Care, Department of Pediatrics, Jordan University Hospital, Amman, Jordan; 10 Department of Radiology, Blacktown and Mount-Druitt Hospital, Sydney, NSW Australia; 11 Department of Pediatrics, Children’s Hospital of Michigan, Detroit, USA

**Keywords:** Respiratory syncytial virus, Infections, Hospitalization, Lower respiratory tract

## Abstract

**Background:**

Respiratory syncytial virus (RSV) is a common cause of acute lower respiratory tract infections, particularly in infants and young children during winter. We aimed to study the demographics and clinical characteristics of RSV infections and age-related patterns.

**Methods:**

This retrospective study evaluated pediatric respiratory syncytial virus (RSV) infections conducted in Jordan from September 2021 to March 2022. Patients under the age of five who had viral polymerase chain reaction results showing RSV infection from nasopharyngeal aspiration were included. In addition, demographic information, medical history, and clinical data were gathered. These included comorbidities, outcomes, length of stay, ICU hospitalization, use of antibiotics, and oxygen supplementation.

**Results:**

A total of 199 patients were included. Most patients were males (56.8%) and less than one year (43.7%). Children aged between 1 and 2 years presented with more shortness of breath (90.1%) than infants and children more than two years (66.7% and 87%, respectively) (*p* < 0.001). Older children (> 2 years) were significantly more likely to use antibiotics and have ICU admission than younger children ≤ 2 years (*p* = 0.045 and 0.018, respectively). There was no relationship between age groups, recurrent hospitalization, previous RSV infection, oxygen therapy, coinfection, and hospitalization duration. The respiratory rate was higher among patients with co-infection (*p* = 0.031).

**Conclusion:**

The current study provides information on the demographics and clinical characteristics of RSV infections. These findings contribute to a nuanced understanding of RSV infections in the specified population, emphasizing age-specific variations and clinical implications for better management strategies.

## Introduction

Globally, respiratory syncytial virus (RSV) is considered a major cause of acute lower respiratory tract infections (ALRI), and the most common cause of bronchiolitis in hospitalized children during winter [1, 2]. In 2019, there were an estimated 33 million RSV infections, 3.6 million RSV ALRI hospitalizations globally, and 101,400 RSV-attributable deaths in children below the age of five [3]. More than 97% of these deaths occurred in low and middle-income countries [3]. A surveillance study of RSV in the Middle East found RSV-associated hospitalization rates were higher than those in some US-based hospitals [4].

Peak rates of RSV infections occur in infants aged six weeks to 6 months, but it can cause infections in all age groups [1]. RSV infections usually present with signs and symptoms of upper and/or lower respiratory tract infections, ranging from rhinorrhea, nasal congestion, and fever to wheezes and respiratory failure [5]. Bronchiolitis is a common manifestation in infants compared to children aged 2–5 years [6]. Viral pneumonia can also occur with bronchiolitis caused by RSV without secondary bacterial infection (6). In 2016, it was found that 22% of patients under the age of two hospitalized for RSV required admission to the intensive care unit (ICU) [7]. Risk factors for severe outcomes can include co-infection, prematurity, chronic lung disease, immunosuppression, congenital heart disease, and co-morbidities (neuromuscular disease, airway anomalies, asthma, or trisomy 21) [5, 8]. However, most RSV-associated admissions occur in healthy infants [6]. Other environmental and host-related factors have been identified as risk factors for a severe outcome for RSV infection, such as low socioeconomic status, male sex, siblings, lack of breastfeeding, daycare attendance, and family history of atopy [9].

It is not uncommon for RSV to be co-detected with other viruses, such as influenza A and B, human rhinovirus (HRV), human enterovirus (HEV), and human metapneumovirus (hMPV) [10]. In most cases, there are no differences in clinical severity in patients with RSV mono-infections and RSV co-infections [11]. One study found RSV-hMPV coinfections might be associated with an increased risk of ICU admission [11]. Another study found RSV-positive children co-infected with other respiratory viruses, mostly HRV, were less likely to present with nasal congestion and sore throat, less likely to require oxygen, but had a more extended hospitalization [10].

The mainstay treatment for RSV is primarily supportive care, such as antipyretics, hydration, and respiratory support [5]. The latter includes humidified oxygen therapy or even endotracheal intubation with mechanical ventilation [5]. In 2023, the US Food and Drug Administration (FDA) approved Nirsevimab, a long-acting monoclonal antibody product, for use in newborns and infants to protect against RSV disease [12]. It is recommended to administer Nirsevimab to all infants who are less than eight months old and are born during or entering their first RSV season [12]. It is a passive immunization; however, it was recommended to include Nisrevimab in the Childhood Immunization Schedule and Vaccines for Children program in the United States [13]. The next steps in RSV control would be the availability of live attenuated vaccines as well as vaccines available globally, particularly in low- or middle-income countries where more than 99% of RSV-associated deaths occur annually [13]. We aim to study how the outcome of RSV infections can be affected by different factors, such as patients’ demographics and clinical characteristics.

## Methodology

### Study design

This was a retrospective study that was conducted in Jordan to evaluate RSV infections among pediatric patients. Data was collected between September 2021 and March 2022. Patients admitted to the hospital and under 5 years old with positive nasopharyngeal aspiration (NPA) with viral polymerase chain reaction (PCR) for RSV were eligible to be included in this study.

### Data collection and measures

The medical records of 199 patients with RSV-positive NPA results were reviewed. Patients’ parents were provided a cover letter, including information about the study and its goals. Data included patient age, gender, gestational age, and measurements. Other data collected included a history of RSV infection, recurrent admissions, and comorbidities. Patients’ signs included respiratory rate, heart rate, and temperature. Details of patients’ course during hospitalization were also collected, such as length of stay, admission to the intensive care unit (ICU), antibiotic use, and oxygen supplementation.

### Nasopharyngeal sampling and viral detection

The study utilized the same PCR tests in both time periods, maintaining consistent testing frequency. Data from multiplex respiratory pathogen real-time PCR tests conducted on nasopharyngeal swab specimens were retrieved from the electronic-based molecular diagnostic laboratory records at Jordan University Hospital (JUH). Nasopharyngeal swabs were collected using Copan media designed for universal transport and preservation and stored at a temperature of -20 °C. Nucleic acid extraction was performed using conventional real-time PCR methods, specifically the FTD Respiratory Pathogens 21 Assay Kit and the EZ 1 and 2 Virus Mini Kit V2.0 by Qiagen. The following viruses were identified: *Respiratory Syncytial Vvirus (RSV), Human Aadenovirus, Human Rhinovirus (HRV), Human Enterovirus (HEV), Human Parainfluenza Viruses 1–4,Hhuman Metapneumoviruses (hMNPV), Influenza A, A/H1N1, and B, Parechovirus, Human Bocavirus (HBoV), as well as Coronaviruses 229E, NL63, OC43, and HKU1.* Additionally, the study also targeted the detection of Plasma Pneumonia, and Hemophilus Pneumonia.

### Statistical analysis

Data was analyzed using Statistical Package for Social Sciences (SPSS) version 23.0 (SPSS Inc., Chicago, IL, USA). Descriptive statistics were used to describe the characteristics of the study sample and continuous data (mean ± SD, frequency, and percentages). The differences between mono-infection and coinfection were explored using the chi-square test. A T-test was used to compare the mean between groups. The variables with statistical significance were included. A P value < 0.05 was considered statistically significant.

### Ethical considerations

The study was conducted according to the guidelines of the Declaration of Helsinki and approved by the Institutional Review Board of Jordan University Hospital (reference number 2021/374). The data’s anonymity and confidentiality were secured by providing each participant with an identity number that was only visible to the research team.

## Results

### Demographic characteristics

The study population’s demographic characteristics are shown in Table 1. Among the 199 patients, most children of the population were male (56.8%), and less than one year old (43.7%). The vast majority of the participants had a term gestational age (*n* = 141, 72.3%), followed by preterm patients (*n* = 51, 26.2%), and less than 5% of participants (*n* = 3, 1.5%) were post-term. The mean weight of participants was (M = 8.58, SD = ± 5.45).

**Table 1:** Demographic characteristics of the study population (*n* = 199)

Demographic characteristics	
Mean Age ± SD (years)	1.306 ± 1.24
Mean Age ± SD (Months)	15.9 ± 15.1
**Age categories**	
Less than one year From 1 to 2 years More than two years	87 (43.7%) 81 (40.7%) 31 (15.6%)
**Gender**	
Female Male	86 (43.2%) 113 (56.8%)
**Gestational age**	
Preterm (< 38) Term (38–40) Post-term (> 40)	51 (26.2%) 141 (72.3%) 3 (1.5%)
Mean Weight ± SD, Kg	8.58 ± 5.45
Mean Height ± SD, Cm	67.45 ± 16.79

### Clinical data

The study population’s clinical characteristics are shown in Table 2. Based on the available retrospective data, it was found that a majority of the patients (93.5%) had no documented history of previous RSV infections The most frequent symptom was cough (*N* = 171, 85.9%), followed by descending order with shortness of breath (*N* = 158, 79.4%), then wheezing (*N* = 76, 38.2%). The mean length of stay (LOS) was 9.3 ± 15.6 days. Among our cohort (199 patients) 92.4% required oxygen therapy and 53.1% were admitted to the ICU. A majority of patients (87.4%) were managed with antibiotics. 31.66% of the infected children had coinfection with other viruses. Rhinovirus was the most frequent type of coinfection (17.1%), while the lowest percentage was (0.5%) for Influenza B, Parainfluenza3, Parainfluenza 4, Hemophilus pneumonia, and parechovirus.

**Table 2:** Clinical characteristics of the study population (*n* = 199)

Clinical parameter	
The vital sign (Mean ± SD)	
Respiratory rate Heart rate Temperature	40.63 ± 15.61 135.54 ± 25.11 36.97 ± 3.05
**Respiratory symptoms n (%) Cough**	
Yes	171 (85.9%)
No	28 (14.1%)
**SOB**	
Yes No	158 (79.4%) 41 (20.6%)
**Wheeze**	
Yes No	76 (38.2%) 123 (61.8%)
**Antibiotic usage n (%)**	
Yes No	174 (87.4%) 25 (12.6%)
**O2 therapy**	
Yes No	183 (92.4%) 15 (7.6%)
**Admission**	
ICU Floor	104 (53.1%) 92 (46.9%)
**Previous RSV infection**	
Yes No	13 (6.5%) 186 (93.5%)
**Comorbidities**	
Yes No	77 (38.7%) 122 (61.3%)
**Recurrent admission**	
Yes No	33 (16.6%) 166 (83.4%)
**Coinfection**	
Yes No	63 (31.66%) 136 (68.34%)
**Mean Hospitalization days ± SD**	9.3 ± 15.6

### Relationship between age groups and clinical characteristics

Regarding the respiratory symptoms at the time of presentation, the analysis demonstrates that children aged between 1 and 2 years presented with more shortness of breath, 90.1%, compared to infants and children more than two years, with a percentage of 66.7% and 87%, respectively (*p* < 0.001) (Table 3). However, other respiratory symptoms like cough and wheezing were statistically insignificant.

**Table 3:** Demographics and clinical characteristics according to age groups

Variable	Less than one year	From 1 to 2 years	More than two years	*P*-Value
	(*n* = 87)	(*n* = 81)	(*n* = 31)	
Mean Age ± SD (years)	0.31 ± 0.23	1.5 ± 0.31	3.4 ± 1.37	**< 0.001**
Mean Age ± SD (Months)	3.83 ± 2.8	18.66 ± 3.8	42.47 ± 16.7	**< 0.001**
**Gender**				0.78
Female	40 (46%)	33 (40.7%)	13 (41.9%)	
Male	47 (54%)	48 (55.3%)	18 (58.1%)	
**Gestational age**				0.87
Preterm (< 38)	24 (27.9%)	20 (25.3%)	7 (23.3%)	
Term (38–40)	60 (69.8%)	58 (73.4%)	23 (76.7%)	
Post-term (> 40)	2 (2.3%)	1 (1.3%)	0 (0%)	
Mean Weight ± SD	6.34 ± 3.88	8.44 ± 3.99	15.25 ± 7.02	**< 0.001**
Mean Height ± SD	59.89 ± 12	67.75 ± 15.44	88.63 ± 13.54	**< 0.001**
**The vital sign (Mean ± SD)**				
Respiratory rate	42.98 ± 13.8	42 ± 17.2	30.3 ± 11.6	**< 0.001**
Heart rate	140 ± 23	135.3 ± 26.4	122.3 ± 22.4	**< 0.001**
Temperature	36.7 ± 3.4	36.9 ± 3.1	37.4 ± 1	0.52
**Respiratory symptoms n (%)Cough**				0.125
Yes	70 (80.5%)	74 (91.4%)	27 (87.1%)	
No	17 (19.5%)	7 (8.6%)	4 (12.9%)	
**SOB**				**< 0.001**
Yes	58 (66.7%)	73 (90.1%)	27 (87.1%)	
No	29 (33.3%)	8 (9.9%)	4 (12.9%)	
**Wheeze**				0.076
Yes	26 (29.9%)	38 (46.9%)	12 (38.7%)	
No	61 (70.1%)	38 (46.9%)	19 (61.3%)	
**Antibiotic usage**				**0.045**
Yes	72 (82.8%)	71 (87.7%)	31 (100%)	
No	15 (17.2%)	10 (12.3%)	0 (0%)	
**O2 therapy**				0.14
Yes	81 (94.2%)	76 (93.8%)	26 (83.9%)	
No	5 (5.8%)	5 (6.2%)	5 (16.1%)	
**Admission**				
ICU	39 (45.3%)	32 (40%)	21 (70%)	**0.018**
Floor	47 (54.7%)	48 (60%)	9 (30%)	
**Previous RSV infection**				0.566
Yes	4 (4.6%)	6 (7.4%)	3 (9.7%)	
No	83 (95.4%)	75 (92.6%)	28 (90.3%)	
**Recurrent admission**				0.077
Yes	10 (11.5%)	14 (17.3%)	9 (29%)	
No	77(88.5%)	67 (82.7%)	22 (71%)	
**Coinfection**				0.883
Yes	27 (31%)	25 (30.9%)	11 (35.5%)	
No	60 (69.0%)	56 (69.1%)	20 (64.5%)	
Mean Hospitalization days ± SD	10.6 ± 22.7	8.7 ± 6	6.7 ± 3.9	0.46

Table 4 illustrates that older children (> 2 years) were significantly more likely to use antibiotics and have more ICU admission than younger children ≤ 2 years, with a p-value of 0.045 and 0.018, respectively.

**Table 4:** Demographics and clinical characteristics according to co-infection

Variable	Yes (*n* = 63)	No (*n* = 136)	*P*-Value
Mean Age ± SD (years)	1.26 ± 1.2	1.32 ± 1.26	0.726
Mean Age ± SD (Months)	15.33 ± 14.77	16.14 ± 15.37	0.726
**Gender**			0.076
Female	33 (52.4%)	53 (39%)	
Male	30 (47.6%)	83 (61% )	
**Gestational age**			0.129
Preterm (< 38)	20 (32.8%)	31 (23.1%)	
Term (38–40)	39 (63.9%)	102 (76.1%)	
Post-term (> 40)	2 (3.3%)	1 (0.7%)	
Mean Weight ± SD	8.63 ± 4.42	8.56 ± 5.87	0.934
Mean Height ± SD	69.59 ± 17	66.51 ± 16.64	0.241
**The vital sign (Mean ± SD)**			
Respiratory rate	44.13 ± 15.19	39.01 ± 15.59	**0.031**
Heart rate	134.76 ± 28.50	135.90 ± 23.46	0.768
Temperature	37.35 ± 0.98	36.78 ± 3.62	0.219
**Respiratory symptoms n (%) Cough**			0.34
Yes	52 (82.5%)	119 (87.5%)	
No	11 (17.5%)	17 (12.5%)	
**SOB**			0.99
Yes	50 (79.4%)	108 (79.4%)	
No	13 (20.6%)	28 (20.6%)	
**Wheeze**			0.51
Yes	22 (34.9%)	54 (39.7%)	
No	41 (65.1%)	82 (60.3%)	
**Antibiotic usage**			0.18
Yes	58 (92.1%)	116 (85.3%)	
No	5 (7.9%)	20 (14.7%)	
**O2 therapy**			0.11
Yes	61 (96.8%)	122 (90.4%)	
No	2 (3.2%)	13 (9.6%)	
**Admission**			0.88
ICU	39 (61.9%)	65 (48.9%)	
Floor	24 (38.1%)	68 (51.1%)	
**Previous RSV infection**			0.94
Yes	4 (6.3%)	9 (6.6%)	
No	59 (93.7%)	127 (93.4%)	
**Comorbidities**			0.078
Yes	30 (47.6%)	47 (34.6%)	
No	33 (52.4%)	89 (65.4%)	
**Recurrent admission**			0.52
Yes	12 (19%)	21 (15.4%)	
No	51 (81%)	115 (84.6%)	
Mean Hospitalization days ± SD	8.28 ± 4.65	9.78 ± 18.7	0.53

Data shows no relationship between age groups, recurrent hospitalization, previous RSV infection, oxygen therapy, coinfection, and hospitalization duration.

### Relationship between coinfection and demographic and clinical characteristics

**Fig. 1:**
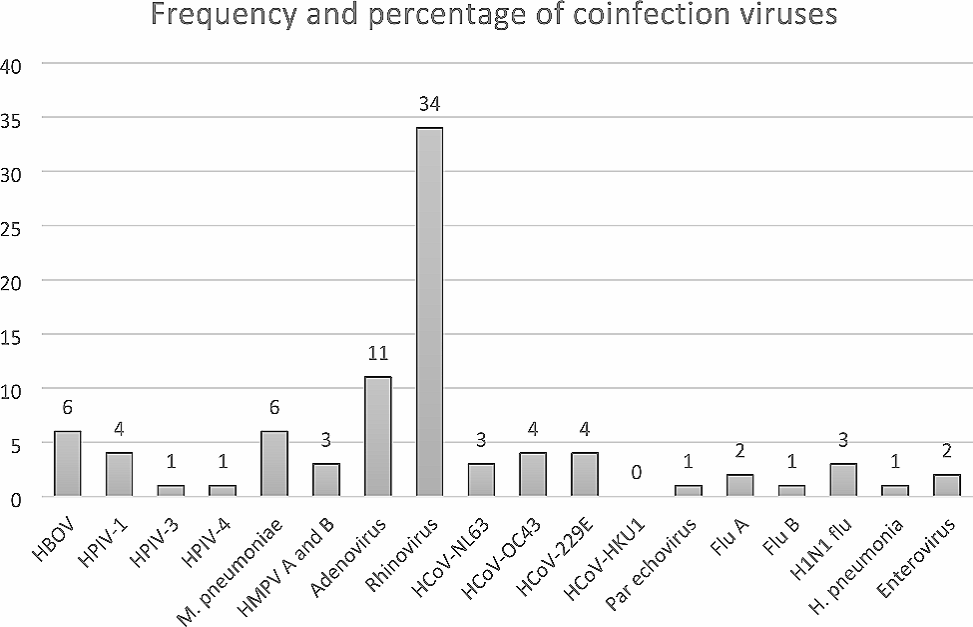
Frequency and percentage of coinfection viruses. Human bocavirus (HBOV), Human parainfluenza viruses (HPIV) 1, 3, and 4, Mycoplasma pneumonia (M. pneumoniae), Human metapneumovirus (HMPV) A and B, Human coronavirus (HCoV) NL63, OC43, 229E and HKU1, Influenza (flu) **A** and B. H1N1 flu, Hemophilus pneumonia (H. pneumonia)

Patients coinfected with other viruses were compared with those infected with RSV alone. There was no statistically significant difference between those two groups regarding demographic and clinical characteristics. However, the respiratory rate was higher among patients with co-infection (*P* = 0.031) (Table 4). Figure 1. Shows the frequency of co-infection viruses.

## Discussion

RSV is a significant cause of respiratory infections in children. This study presents the demographic and clinical characteristics of children affected with RSV. In terms of demographics, there was a predominance of male children in the study population, which aligns with previous literature suggesting a potential gender susceptibility to RSV [8, 14, 15]. The majority, being less than one year old, emphasizes the vulnerability of infants to RSV, reflecting a common pattern in RSV epidemiology [6]. In our study, the majority affected by RSV were term gestational age and had no co-morbidities. RSV is a heavy burden to children, families, and the health system since it is shown that the majority of those affected and hospitalized are healthy and full-term [1, 6]. Our study’s mean length of stay (LoS) of 9.3 days reflects RSV’s substantial burden on healthcare resources. The recent approval of nirsevimab is particularly significant, as it is eligible for administration in healthy infants under eight months old, potentially decreasing healthcare visits and hospitalizations [13]. The availability and approval of Nirsevimab globally, specifically in low and middle-income countries, could potentially contribute to lowering RSV-related mortality in those regions.

In the present study, over 93% who were admitted to the general inpatient unit or ICU did not experience prior exposure to RSV. This suggests that a history of RSV infection may provide some immunity, which could prevent severe symptoms that may require admission. Two studies reported similar findings, where previous RSV infection is effective in giving short immunity for several months, reducing the risk of severe reinfection and minimizing its duration [16, 17].

Like other lower respiratory infections, the most frequent symptoms in RSV-infected children were cough, shortness of breath, and wheezing. The high percentage of patients requiring oxygen therapy and ICU admission highlights the severity of RSV infections. In a study conducted by Khouri et al., it was found that RSV-positive children are more likely to require oxygen [10]. When comparing age groups and clinical characteristics, our study found patients over the age of one presenting with more shortness of breath than infants. We also found that 70% of our patients over the age of 2 years required ICU admission, which was more significant than patients under 2 years. Older children have a higher likelihood of having co-morbidities, which can increase the risk of severe RSV. In general, RSV globally causes significant morbidity and mortality burden in children 0–5 years old, especially in the first six months of life and in low- or middle-income countries [3].

Our study found that a third of the patients had co-infections. Khouri et al. highlighted that RSV-positive subjects co-infected with other respiratory viruses presented less frequently with nasal congestion and sore throat [10]. In most cases, there are no differences in clinical severity in patients with RSV mono-infections and RSV co-infections [11]. We found no statistical difference when comparing mono-RSV infection and co-infections except in respiratory rate, which was elevated in co-infections. Of the 199 children in our study, around 174 (87.4%) of RSV-infected patients were given antibiotics. This rate exceeds the rates of antibiotic prescriptions found in the USA, Canada, and Europe, which is only around 40–45% [18, 19]. Previous literature suggests that bacterial co-infections in RSV infections are generally considered rare, with one study reporting a prevalence of 1.2% [18]. However, recent studies by Thorburn et al., Ghazaly et al., and Yang et al. have revealed higher rates of bacterial co-infections, particularly in severe RSV cases requiring intensive care unit (ICU) admission and intubation [20–22]. The high rate of antibiotic use among our cohort despite only 50% required PICU admission may be attributed to the lack of routine laboratory diagnosis of viral infection in Jordan, which can lead to antibiotics administration for respiratory infections in children, or due to the haphazard antibiotics prescription similar to other developing countries [23]. Additionally, results from a nasopharyngeal aspirate (NPA) at institutions offering this test may be available within one to a couple of days. Furthermore, a recent report by Hamdan et al. discusses the patterns of antibiotic use in children hospitalized with RSV in Amman. The report highlights that clinicians in developing countries, where testing for common respiratory viruses is unavailable, rely on clinical presentation and judgment to differentiate between viral and bacterial infections [24].

A limitation of the study was a small study population. Our study included one center; we would suggest a multi-center study throughout Jordan to get more conclusive surveillance.

## Conclusion

This study presents a comprehensive analysis of the demographic and clinical characteristics of pediatric cases infected with Respiratory Syncytial Virus (RSV). The vulnerability of infants is underscored by the majority of cases occurring in those less than one year old. Notably, RSV affects primarily healthy, full-term children with no co-morbidities, highlighting the significant burden it imposes on this population. The study reveals a substantial mean length of stay, indicating RSV’s considerable impact on healthcare resources. While a history of RSV infection may confer some level of immunity and potentially prevent severe symptoms requiring admission, a concerning finding is the high rate of antibiotic use, emphasizing the importance of judicious antibiotic usage in pediatric respiratory infection management. The study provides valuable insights into RSV infections in Jordan, emphasizing the ongoing necessity for preventive measures, widespread vaccine access, and improved strategies to address RSV’s considerable impact on children globally.

## Data Availability

The data from the present research are accessible from the corresponding author upon reasonable request.
